# Thematic analyses of participant survey responses following dermatology ECHO programs with dermoscopy: Practical tips and lessons learned

**DOI:** 10.3389/fdgth.2023.1163556

**Published:** 2023-03-22

**Authors:** T. Austin Black, Joshua R. Parbs, Anthony J. Teixeira, Peggy Cyr, Kelly C. Nelson, Henry Stoddard, Elizabeth V. Seiverling

**Affiliations:** ^1^ John P. and Katherine G. McGovern Medical School, University of Texas Health Science Center at Houston, Houston, TX, United States; ^2^ Tufts University School of Medicine, Boston, MA, United States; ^3^ Davidson College, Davidson, NC, United States; ^4^ Maine Medical Partners, Department of Family Medicine, Portland, ME, United States; ^5^ Department of Dermatology, The University of Texas MD Anderson Cancer Center, Houston, TX, United States; ^6^ Center for Interdisciplinary Population and Health Research, MaineHealth Institute for Research, Portland, ME, United States; ^7^ Department of Dermatology, Tufts University School of Medicine, Boston, MA, United States

**Keywords:** dermoscopy, medical education, Project ECHO, telementoring, skin cancer, education

## Abstract

**Introduction:**

Skin cancer is a major public health concern in the United States, reflecting approximately one in every three cancer diagnoses. Despite the high incidence of skin cancer, access to dermatologists is limited, especially in rural areas. Primary care physicians play a pivotal role in the evaluation of skin conditions, but dermatology training gaps exist in primary care training programs.

**Objectives:**

This study examines the use of the Project ECHO (Extension for Community Healthcare Outcomes) knowledge-sharing framework to provide dermoscopy and skin cancer detection training to primary care providers (PCPs).

**Methods:**

Responses to surveys administered to participants in two separate dermoscopy-focused Project ECHO courses were analyzed. Survey responses were collected over a 4-year period for the two courses, which were delivered in Maine and Texas. Thematic analysis of the qualitative data was performed, revealing codes and subcodes that indicated several overall trends.

**Results:**

Overall, most respondents indicated the ECHO sessions to be helpful, reporting an increase in confidence and knowledge in dermoscopy. Other codes reflected a positive reception of the learning materials and teaching styles. Furthermore, participant survey analyses highlighted areas of improvement for future ECHO course sessions.

**Conclusions:**

This thematic analysis of Project ECHO courses in dermatology with dermoscopy demonstrates the feasibility of using virtual educational platforms to effectively teach PCPs about dermoscopy and skin cancer, with high levels of participant satisfaction. The need to keeping the educational sessions brief, avoid scheduling sessions on high-volume patient care days, and provide a means for participants to obtain hands-on training in the operation of a dermatoscope were among the top lessons learned.

## Introduction

The World Health Organization (WHO) estimates that one in every three cancers diagnosed is skin cancer (1). While examinations to detect skin cancer are noted to be among the most effective screening interventions in medicine, skin cancer incidence continues to increase (2–4). Performing skin examinations is an integral part of a dermatologist's practice, yet many regions of the United States have no dermatologists (5). In these regions, primary care providers (PCPs) may be a critical resource in the diagnosis of skin cancers. However, as many as 89% of PCPs cite a lack of dermatologic training as a barrier to performing skin examinations (6).

Two distinct yet quite similar educational interventions were developed to combat this barrier by providing PCPs with foundational skin cancer detection training. The two interventions were similar as they both taught an algorithm-based approach to dermoscopy (7). A dermatoscope is a low-cost hand-held medical instrument that pairs a 10× magnifying lens with a polarized light source. With appropriate training, use of a dermatoscope reduces the rate of both false positives and false negatives in skin cancer detection examinations (8).

Both training interventions used the evidence-based Project ECHO (Extension for Community Healthcare Outcomes) knowledge-sharing framework (9). The courses were distinct in their target geographical areas and end-users. Course “A” was delivered throughout the state of Maine, targeting practicing PCPs in rural counties. Highlighting these rural areas was a key component of this study, given that Maine has fewer than 3 dermatologists per 100,000 people (5). This ratio is among the lowest in the United States, and thus the importance of dispensing this knowledge to PCPs in the state is particularly high. Course “B” was delivered to resident physicians with the Texas Tech University Health Sciences Center Department of Family and Community Medicine in El Paso, TX.

In this study, we conducted a thematic analysis of 474 course-specific survey responses collected over the course of 4 years to determine both the strengths of the courses and areas for possible improvement. In this article, we also summarize best practices, practical tips, and lessons learned that may benefit others developing interactive virtual educational interventions for dermoscopy and skin cancer.

## Methods

Both interventions received Institutional Review Board approval, and participants reviewed a statement of consent and agreed to participate in the study. Each participant had the opportunity to opt out of data collection at any time. We then extracted data on programmatic outcomes collected from participants in the two Project ECHO telementoring educational interventions.

Responses were collected in the form of a survey with open- and closed-ended questions; however, this analysis included only the responses to open-ended survey questions. Responses were collected from September 2019 to May 2022. The following data fields were included, addressing qualitative aspects of each participant's experience as outcomes: identifying strengths and weaknesses of each intervention; defining how the participant would change their clinical practices based on exposure to the intervention; and explaining why they participated in the intervention sessions. Participants were also invited to share general comments on the intervention.

All participants were sent a URL via email that they could use to access the survey at the end of the ECHO session. Response rates were assessed to ensure validity. Participants included resident and attending physicians from academic and community health centers, nurse practitioners, and physician assistants who signed up to participate in the Project ECHO intervention. We included all pertinent responses from participants in each of the sessions in the analysis. Responses were analyzed using a descriptive approach to inquiry, identifying themes and patterns from the data rather than creating codes *a priori*.

**Figure 1:**
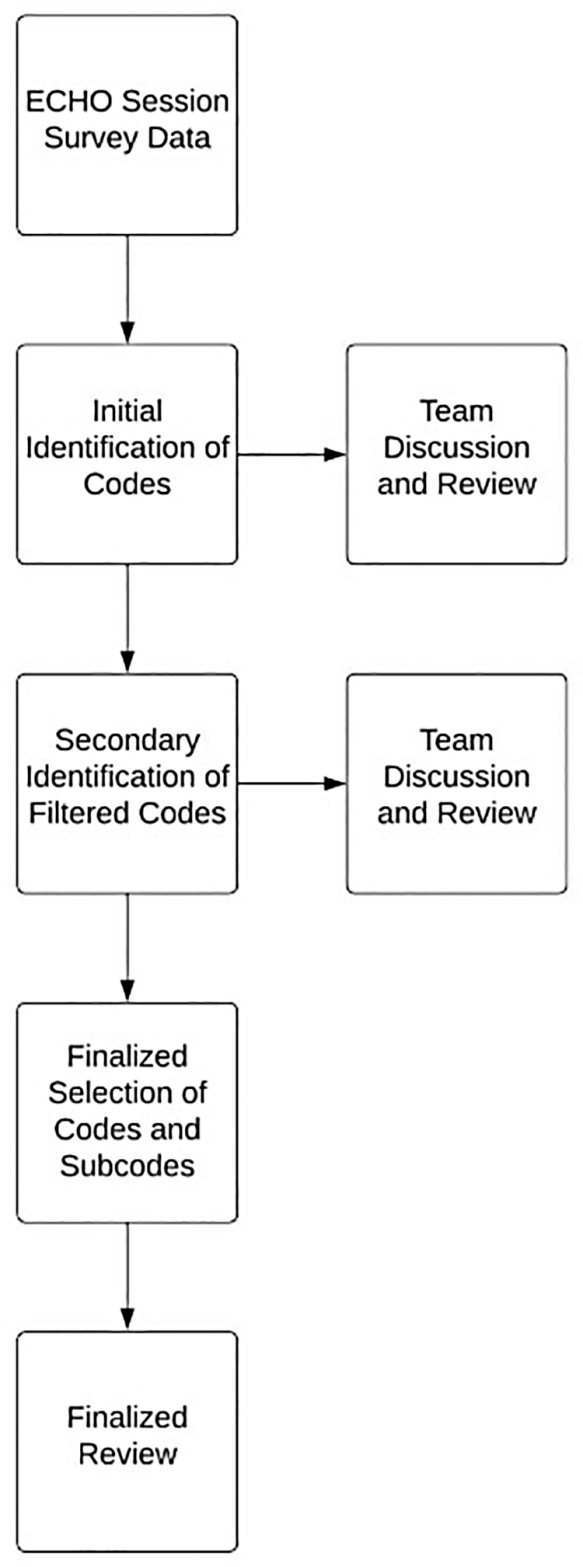
Flow chart illustrating the stepwise thematic analysis process.

We used thematic analysis to identify and investigate trends in the data collected from 2019 to 2022 on the programmatic outcomes of the two interventions. Three team members (TB, JP, and AT) independently reviewed and condensed deidentified survey data to identify general codes and subcodes (Figure 1). These initial codes were discussed with the larger group and modified based on feedback, generating a secondary set of filtered codes. The final rounds of data review and coding generated the finalized set of codes and subcodes presented in this paper. The finalized codes were discussed in the larger group to ensure face validity.

Once the finalized codes were determined, the data were analyzed to determine the participant-level frequency of each thematic element by ECHO site location. Exemplar quotes were identified to further support the validity of the final codes and to best reflect the overall dataset (Table 1). Data were collected using Smartsheets, data analysis was conducted using Microsoft Excel (Version 2206), and figures were created using R (version 4.0.2) with the ggplot2 package (10).

**Table 1:** Themes and subthemes for each qualitative survey question.

Themes and subthemes					
Survey questions	Theme	*Subtheme*	Frequency		
What did you like best about the program?	1–Likes		34	5	39
		1.1–*Examples and practice questions*	17	42	59
Additional comments		1.2–*Conciseness and presentation*			
What did you like least about the session?	2–Dislikes/areas for improvement				
Is there anything that we could change for the program next year?		2.1–*Nothing*	11	144	155
Is there anything you would suggest to improve today's call?		2.2–*Sessions were too long/went over time*	5	1	6
What additional dermatology topics would you like to see offered?		2.3–*Disliked sessions on Monday*	0	10	10
Additional comments		2.4–*Disliked use of Zoom/had technical difficulties*	5	17	22
		2.5–*Desire for additional content*	5	96	101
		2.6–*Hands-on dermatoscope practice*	3	5	8
What are the primary reasons you participated in this training?	3–Motivations for participating				
		3.1–*General interest in dermatology*	0	26	26
		3.2–*Requirement of residency program*	0	5	5
		3.3–*Gain or increase dermoscopy proficiency*	0	36	36
		3.4–*Learning more about the role of a PCP in dermatology*	0	24	24
		3.5–*Improve patient care*	0	23	23
		3.6–*Desire for improvement*	0	64	64
		3.7–*High-quality instruction*	0	14	14

## Results

The themes, subthemes, and frequency of each subtheme are presented in Table 1. Table 2 provides examples of notable responses coded under each subtheme. In aggregate, 592 responses were coded: 98 under Theme 1 (likes), 302 under Theme 2 (dislikes), and 192 under Theme 3 (motivations for participating).

**Table 2:** Subtheme exemplar responses.

Notable responses	
Subtheme	Response
*1.1–Examples and practice questions*	
	“I thoroughly enjoyed the examples that were given and being able to see different versions of the same pathology.” “I like the questions at the end as it helps me to understand different approaches for biopsy. Also it was nice to see examples so I can understand the patterns of different lesions.” “I really enjoyed and learned a lot from the interactive cases.”
*1.2–Conciseness and presentation*	
	“I like the 1 hour sessions that were interactive like this. Much more engaging.”
	“Quick, well-organized, clear instruction.”
*2.1–Nothing*	
*2.2–Sessions were too long/went over time*	
	“More one hour sessions instead of the long 2 hour sessions or at least make time for breaks during the 2 hour sessions.”
*2.3–Disliked sessions on Monday*	
	“It is hard to fit in an ECHO mid-day, esp. on Mondays. I got to this one late due to patient care.”
*2.4–Disliked use of Zoom/had technical difficulties*	
	"If only it was in person; however, virtually is the next best thing.” “I had trouble with Zoom on my end, so I ended up missing a portion of the presentation.”
*2.5–Desire for additional content*	
	“More rashes as opposed to moles/skin cancer.”
*2.6–Hands-on dermatoscope practice*	
	“Need hands-on experience.”
*3.1–General interest in dermatology*	
	“I am deeply interested in all things dermatology.”
*3.2–Requirement of residency program*	
*3.3–Gain or increase dermoscopy proficiency*	
	“Improve ability to differentiate between benign and potentially malignant lesions using dermoscopy.” “To get better at using dermoscopy and identifying lesions appropriate for dermatology referral, biopsy, etc.”
*3.4–Learning more about the role of a PCP in dermatology*	
	“Interest in dermatology in the primary care setting because of shortage of dermatologists in the area.”
*3.5–Improve patient care*	
	“To improve the quality and specificity of the care I provide my patients and to refer more appropriately.” “I am asked to care for patients with conditions like these regularly.”
*3.6–Desire for improvement*	
	“To better differentiate benign versus possible malignancy and to help to limit unnecessary biopsies when able.”
*3.7–High-quality instruction*	
	“I’m not currently in practice but have learned a lot from the two sessions I’ve attended and appreciate the opportunity to keep up with the latest techniques and recommendations for diagnosis and treatment. Presentations are very well made.” “Great learning and great teaching. Super helpful in primary care.”

**Figure 2:**
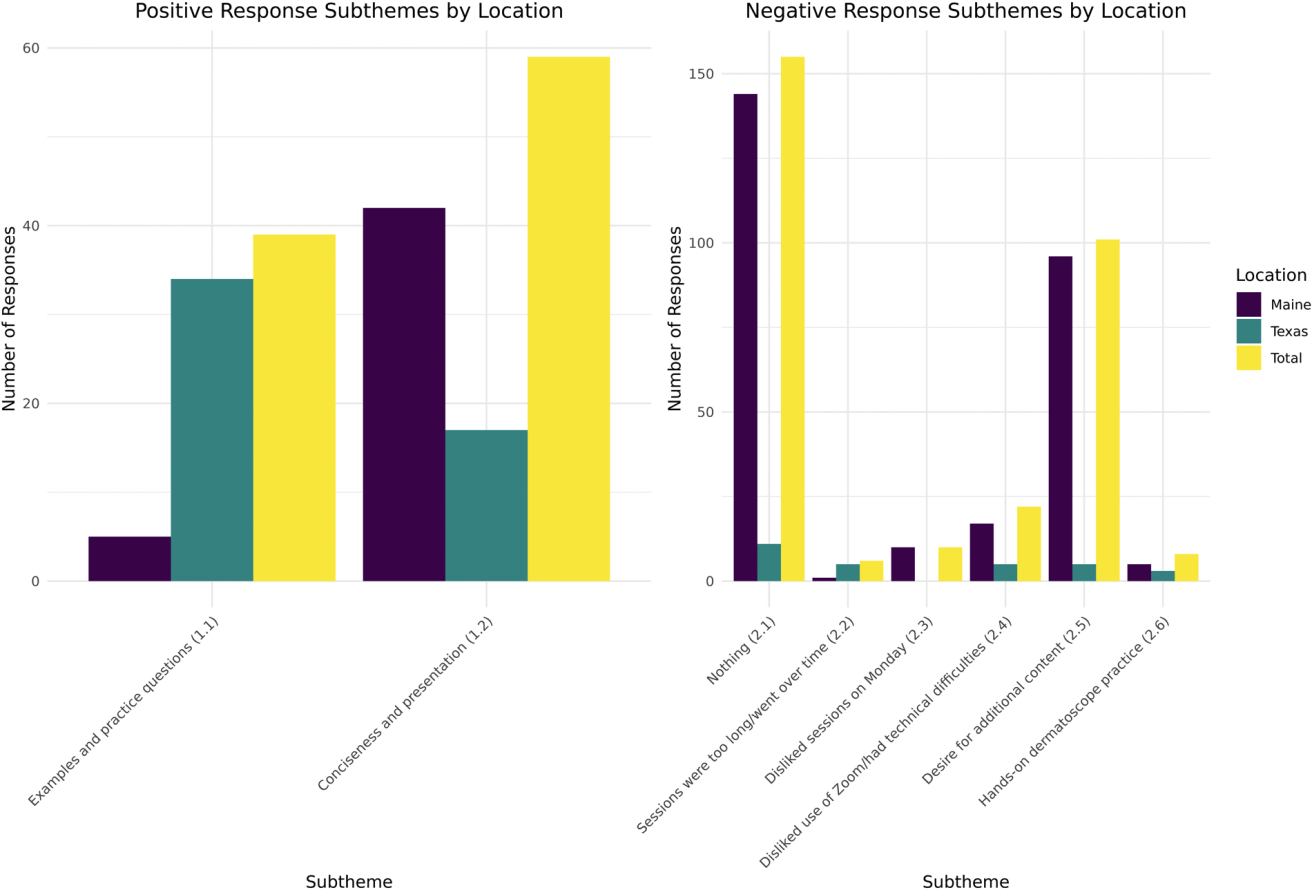
Results relating to Theme 1 (likes) and Theme 2 (dislikes/areas for improvement) by location.

Results from both site locations for Theme 1 (likes) indicated that the participants’ favorite aspect of the program was the concise nature of the 1-h presentations followed by the course-integrated practice questions and examples. When the responses were separated by location, the concise nature of the presentation was favored in Maine, while the practice questions and examples were favored in Texas. Figure 2 displays the results pertaining to Theme 1 (likes) in graphical form.

Questions addressing Theme 2 components (dislikes) elicited a 47% response rate; 51% (*n* = 155) of these responses indicated no dislikes or areas for improvement. The most commonly identified area for improvement related to a desire for additional content, as solicited by the question, “What additional dermatology topics would you like to see offered?” Technical difficulty was the second-most common dislike, with increased frequency noted during the initial years of the SARS-CoV-2 pandemic. In Maine, 88% (*n* = 15) of responses mentioning Zoom (Zoom Video Communications, Inc., San Jose, CA) or other technical difficulties occurred in the 2019 and 2020 surveys, and in Texas, all the responses relating to technical difficulties occurred in the 2020–2021 year. Finally, multiple responses indicated that sessions on Mondays, and sessions longer than one hour, were dispreferred. Figure 2 displays the results pertaining to Theme 2 (dislikes) in graphical form.

**Figure 3:**
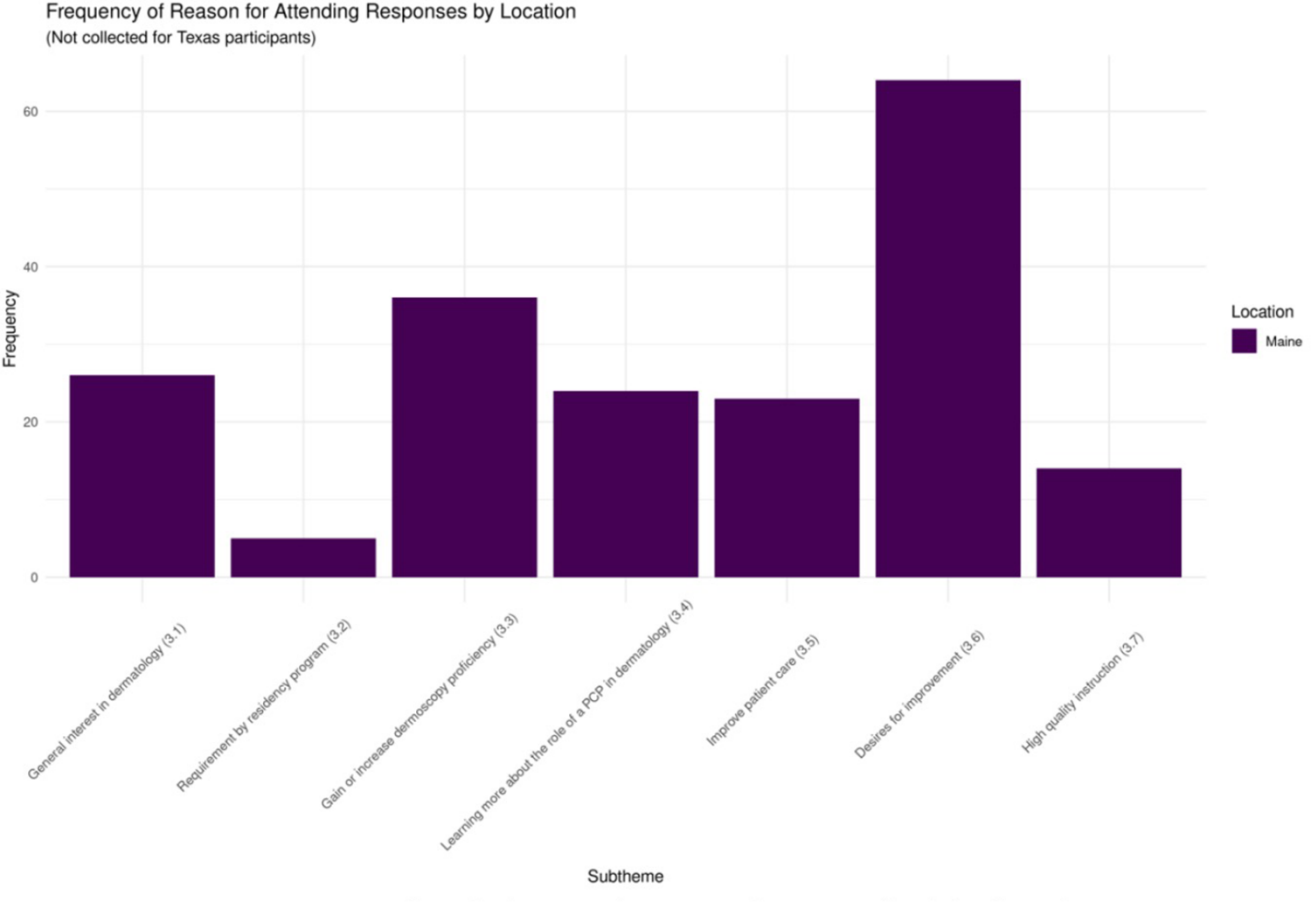
Results relating to Theme 3 (reasons for attending) by location.

There was a 78% response rate for the question relating to Theme 3 (motivations for participating). The primary sources of motivation for participating were desire for improvement (33%, *n* = 64) and desire to gain or increase proficiency with dermoscopy (19%, *n* = 36). Other popular motivations included a general interest in dermatology (14%, *n* = 26), desire to learn about the role of PCPs in dermatology (13%, *n* = 24), and desire to improve patient care (12%, *n* = 23). Figure 3 displays the results pertaining to Theme 3 (motivations for participating) in graphical form.

Demographic data were collected for participants in the Maine ECHO programs. In the 2019–2020 ECHO program, there were 63 participants: 78% were PCPs, 9% were NPs or PAs, and 13% were dermatologists or ancillary medical staff. There was an average of 27 participants per session. In the 2020–2021 ECHO program, there were 81 participants: 61% were PCPs, 33% were NPs or PAs, and 6% were dermatologists or ancillary medical staff. There was an average of 30 participants per session. Finally, in the 2021–2022 ECHO program, there were 86 participants: 56% were PCPs, 36% were NPs or PAs, and 8% were dermatologists or ancillary medical staff. There was an average of 33 participants per session. The number of participants and average attendance increased in each consecutive year. Demographic data were not available for the El Paso, Texas ECHO programs.

## Discussion

Our data on programmatic outcomes can inform best practices for educational interventions leveraging the Project ECHO telementoring framework. Positive feedback regarding session duration was provided when the sessions were 1 h long, while negative feedback was given when the sessions lasted 2 h or longer. Thus, 1 h is the preferred session duration. Sessions should also be spaced out, potentially monthly, to increase knowledge retention (11). For sessions lasting more than 1 h, breaks at the hour mark are helpful to decrease the fatigue associated with online learning.

Monday was frequently cited as an inconvenient day for a telementoring session. Evidence suggests that healthcare providers’ offices experience the highest demand for appointments and handle the largest volume of patients on Mondays (12, 13). We suggest holding ECHO sessions on Wednesdays, as healthcare offices have been found to see fewer patients on Wednesdays than Mondays, suggesting that PCPs may be less busy (13). If sessions must be conducted on Mondays, recording the session can provide asynchronous access.

Based on the responses under subtheme 1.1 (“Examples and practice questions”), ECHO sessions should include numerous practice questions and examples. Many responses indicated that these practice questions helped clinicians to establish a visible pattern, enabling them to diagnose skin lesions more accurately.

While this qualitative thematic analysis found that course participants value the program for its ability to increase dermoscopy skills and confidence surrounding skin cancer diagnosis, educational interventions do not always translate to changes in practice or improved patient outcomes. However, following nearly 2 years of provision of this Project ECHO dermatology course in Maine, there was a 10% (*p* < .0001) shift from benign to malignant biopsy results in the primary care setting (14). Furthermore, there was a meaningful reduction in the number of patients needing to undergo biopsy to detect skin cancer (14, 15).

There was a strong desire for additional content, as 22% of participants (*n* = 96) responded to the question “What additional dermatology topics would you like to see offered?” This question facilitates improvement of the program by highlighting participants’ current knowledge gaps. We recommend including this question on feedback surveys for all ECHO programs, as the topics mentioned can be incorporated into the following year's curriculum, improving participants’ satisfaction with the course and eliminating knowledge gaps.

For reference, the title of (and thus the topic covered in) each ECHO lecture was as follows: Dermoscopy Basics: Benign Skin Growths; Dermoscopy Basics: Skin Cancer; Basal Cell Carcinoma: Building on Triage Amalgamated Dermoscopic Algorithm (TADA); Nevus vs. Melanoma; Demystifying Skin Biopsies; Psoriasis and Scaly Rashes; Common Skin Infections; Dermatopharmocology; and Dermoscopy Recap.

The lack of responses indicating technical and Zoom-related difficulties in 2021 and 2022 suggests that the use of a virtual setting for ECHO sessions is becoming more feasible and efficient. The virtual setting has multiple advantages, including the ability to teach PCPs from a distance. However, it should be noted that the lack of hands-on experience associated with virtual learning was repeatedly mentioned by respondents. We recommend combating this issue by partnering with a PCP site champion. The Maine ECHO established site champions at each of its ECHO sites. These site champions received training on operation of a dermatoscope, and all of their clinics were equipped with dermatoscopes. Site champions stimulate engagement in the ECHO program and offer in-office, hands-on practice, allowing participants to refine their skills and better achieve the desired program outcomes.

An understanding of participants’ motivations for enrollment prior to conducting the telementoring sessions could be of value; thus, we recommend asking the question “What are your primary reasons for participating in this training?” before the program starts via a precourse survey. With an understanding of the respondents’ motives for participating, the course leaders can better orient their teaching style and the session content toward the participants’ desires, which would likely result in a further increase in satisfaction with the course.

In our study, subthemes 3.4 (“Learning more about the role of a PCP in dermatology”) and 3.5 (“Improve patient care”) are not only applicable to dermoscopy but also generalizable to all ECHO programs in dermatology. As a result, we recommend emphasizing the role of PCPs in dermatology in the delivery of such sessions, such as when to refer to a dermatologist and how to improve patient care.

In summary, our analysis demonstrates high engagement and satisfaction with virtual dermoscopy training delivered via Project ECHO. By incorporating our recommendations, future ECHO programs might achieve improved levels of satisfaction and outcomes. This platform is easily generalizable to all dermatologic topics, and if implemented more widely, Project ECHO may help address the need for more dermatologic care (specifically, skin cancer detection) and training in the primary care setting.

## Conclusion

As the need for dermatologic care throughout the country continues to increase, the delivery of virtual educational interventions offers a method to combat gaps in dermatology training. This analysis found that dermoscopy and skin cancer training delivered via the Project ECHO platform is well received. Optimizing partnerships between PCPs and dermatologists has the potential to allow for the transfer of knowledge, rather than referral of patients, for many common skin conditions, including skin cancer.

## Data Availability

The raw data supporting the conclusions of this article may be made available by the authors on a case-by-case basis. Requests to access the datasets should be directed to Elizabeth V. Seiverling, vseiverling@gmail.com.
